# Mood instability: significance, definition and measurement

**DOI:** 10.1192/bjp.bp.114.158543

**Published:** 2015-10

**Authors:** M. R. Broome, K. E. A. Saunders, P. J. Harrison, S. Marwaha

**Affiliations:** **M. R. Broome**, PhD, MRCPsych, Department of Psychiatry, University of Oxford, Oxford, Oxford Health NHS Foundation Trust, Oxford and Division of Mental Health and Wellbeing, Warwick Medical School, University of Warwick, Coventry; **K. E. A. Saunders**, DPhil, MRCPsych, **P. J. Harrison**, DM, FRCPsych, Department of Psychiatry, University of Oxford, Oxford; **S. Marwaha**, PhD, MRCPsych, Division of Mental Health and Wellbeing, Warwick Medical School, University of Warwick, Coventry and Early Intervention Service, Swanswell Point, Coventry, UK

## Abstract

Mood instability is common, and an important feature of several psychiatric disorders. We discuss the definition and measurement of mood instability, and review its prevalence, characteristics, neurobiological correlates and clinical implications. We suggest that mood instability has underappreciated transdiagnostic potential as an investigational and therapeutic target.

Mood instability is a common experience. In the Adult Psychiatric Morbidity Survey (APMS) 2007 (*n* = 7403),^1^ a population rate of 13.9% was found. It was more common in women than men, peak prevalence was in those aged 16–24, and it gradually declined with age with 7% of 65- to 74-year-olds reporting unstable mood.^1^ Mood instability is reported in 40–60% of those with depression, anxiety disorder, post-traumatic stress disorder and obsessive–compulsive disorder, and is associated with increased health service use and suicidal ideation, independent of neurotic symptoms, alcohol misuse, borderline personality disorder and other confounders.^2^ Complementing these data in the general population, mood instability has clinical significance beyond simply being a feature of several psychiatric disorders: it is involved in their origins and affects prognosis. Mood instability is part of the prodrome of bipolar disorder,^3^ and can occur in the earliest phases of attention-deficit hyperactivity disorder and depressive disorder. It is not only a mediating factor in the pathway from trauma to emerging borderline personality disorder but also in the genesis of hallucinations, paranoia and psychotic disorders.^4^ Mood instability independently predicts worse long-term outcome in euthymic patients with bipolar disorder.^5^

## Defining and measuring mood instability

These findings together indicate that mood instability is an important aspect of psychopathology, and suggest that better understanding of its nature, origins, correlates and implications would be valuable. However, attempts to make progress must first address the lack of consensus as to the definition of mood instability. For example, the APMS studies relied upon a single question to assess mood instability (‘Do you have a lot of sudden mood changes’ with the timescale being ‘suffered this symptom over the last several years’), taken from the structured interview for the DSM-IV diagnosis of borderline personality disorder,^6^ whereas assessments of mood instability in clinical samples typically use one of several rating scales. Clearly, these will result in very different estimates of prevalence and, potentially, in the concept being measured.

The nomenclature surrounding affect or mood includes valence, intensity, frequency of shift, rapidity of rise-times and return to baseline, reactivity to psychosocial cues and the extent to which there is overdramatic expression. The literature spans psychiatry, psychology and neuroscience, and multiple terms are used to describe the same, or related phenomena, including affective instability, emotional dysregulation, mood swings, emotional impulsiveness and affective lability. Collating the main overlapping dimensions, definitions, and their measurement scales, a recent systematic review proposed that mood instability is ‘rapid oscillations of intense affect, with a difficulty in regulating these oscillations or their behavioural consequences’.^7^ The presence of these various elements mandates a multidimensional approach to assessment of mood instability. The current uncertainties may contribute to the fact that patients experiencing unstable mood are not consistently given an explanation by their clinician, even though they value one.^8^

Progress in defining and measuring mood instability can be facilitated by taking advantage of two recent developments. First, the mood instability literature to date is almost entirely derived from the use of retrospective questionnaires. Clearly, this has been and remains a valuable approach. However, responses to retrospective questionnaires are known to suffer from limitations including recall bias, and may be a particular problem for studies of mood instability given its dynamic nature;^9^ both variation and intensity need to be recalled in addition to the mood state *per se*. Momentary assessment and remote monitoring methodologies can largely overcome these problems, and give greater insight and a more detailed quantitative characterisation of the nature of mood instability in daily life. For example, high-frequency prospective automated mood monitoring such as that used in the True Colours system (www.truecolours.nhs.uk) reveals a complex picture in bipolar disorder in which chronic mood instability is more common than discrete episodes.^10^ Second, it is increasingly possible to use remote sensors and other devices (such as via smartphones, smartwatches or patches) to capture the behavioural, physiological and environmental correlates of mood instability, and in this way provide a much richer and deeper understanding.^11^

## Investigations and implications of mood instability

Despite the complexities, mood instability merits greater clinical and research attention than simply being an acknowledged but often neglected component of psychopathology.

### What is its origin?

Mood instability presumably shares some of the same genetic and environmental risk factors as the disorders in which it is a feature but may also have its own causal factors. Longer-term epidemiological studies of mood instability, examining persistence from childhood, will enable a greater understanding of how far it is a precursor to, or a risk factor for, a particular disorder or for multiple outcomes. In addition discovery of causes, trajectories and insights into the nature of mood instability in different disorders would shed light on its shared elements or transdiagnostic aspects. In this regard, mood instability fits well as a construct of the kind envisaged in the Research Domain Criteria (RDoC):^12^ it is likely to reflect problems in a core behavioural function of the brain, seems likely to be related to a dysfunction in neural circuits, and is dimensional.^13^

### What are its cognitive and neural correlates?

Cognitive function is impaired in diagnostic groups where mood instability is prominent^14^ and the relationship between mood instability and fluctuations in attention, cognition, and the underlying brain processes will be of interest. The amygdala and its functional connectivity may be altered,^13^ but the circuits underlying mood stability, and how these are impaired in those with unstable mood remain to be identified. For example, there may (or may not) be a relationship between neural stability (for example in oscillatory activity) and stability as manifested at the cognitive or emotional level. These questions can be investigated by multimodal study of participants with differing degrees of mood instability, including brain imaging methods such as magnetoencephalography.

### What are the implications for treatment?

We can see two facets to this important issue. First, stabilisation of mood could be an early marker or predictor of the subsequent efficacy of interventions to treat clinical mood episodes. This would be analogous to the discovery that the clinical efficacy of antidepressants can be predicted by their acute effects on emotional processing.^15^ If so, it would provide an experimental medicine model for bipolar disorder, as well as for borderline personality disorder and other disorders and prodromal states in which mood instability has a key role, and would facilitate more rapid and cost-effective testing of novel interventions and thereby encourage more innovation and investment in these neglected areas. This approach can in principle apply to psychological as well as pharmacological treatments. Second, stabilisation of mood may be therapeutically valuable beyond the usual sense of preventing episodic relapses in bipolar disorder, given the evidence that unstable mood during euthymia, and in the prodrome of illness, predicts worse functioning and outcome. As such, mood instability may be an important target for early intervention in a variety of conditions. Clearly, it would be necessary to demonstrate the efficacy, safety and acceptability of any such interventions before they could be advocated in clinical practice.

All these investigations of mood instability will be facilitated by the rapid developments mentioned earlier in methods for the more fine-grained and remote assessment of mood and its integration with environmental and physiological data. One result may be the delineation of subgroups based on their mood instability and associated characteristics, which cut across existing syndromal boundaries and which can be tested for their prognostic and therapeutic correlates. A second result may be the identification of mood instability signatures in individual patients that predict an imminent event (such as a manic episode) and which could be rapidly relayed back (for example, via a text to their smartphone), allowing the patient to implement appropriate behavioural changes, or to take additional medication. The feasibility and clinical utility of this approach and technology will need to be carefully tested.

In summary, mood instability is a common, clinically important phenomenon, with functional consequences. More precision in its definition and measurement, in combination with contemporaneous assessment of physiology, behaviour and environment, will facilitate study of its characteristics, correlates and implications. The latter include the possibility that mood instability has a broader potential as a therapeutic target than is currently the case.
